# Opposing end-node and junction-node remodeling patterns in diabetic patients on optical coherence tomography angiography

**DOI:** 10.1038/s41598-026-50339-y

**Published:** 2026-04-25

**Authors:** Mohsen Zare, Saeed Karimi, Mohammad Shojaeinia, Hamid Safi, Homayoun Nikkhah, Seyyed Morteza Hosseini Imeni

**Affiliations:** 1 Department of Ophthalmology, Torfeh Medical Center, Shahid Beheshti University of Medical Sciences, Tehran, Iran; 2 Department of Health Information Technology and Management, School of Allied Medical Sciences, Shahid Beheshti University of Medical Sciences, Tehran, Iran; 3 Ophthalmic Research Center, Research Institute for Ophthalmology and Vision Science, Shahid Beheshti University of Medical Sciences, Tehran, Iran; 4 Department of Ophthalmology, Babol University of Medical Sciences, Babol, Iran

**Keywords:** Diabetic retinopathy, Optical coherence tomography angiography, Vascular network remodeling, Vascular network topology, Vessel skeleton density, Fractal dimension, Biomarkers, Diseases, Endocrinology, Medical research

## Abstract

This cross-sectional study aimed to investigate macular vascular network topology in diabetic patients. We analyzed 3 × 3 mm OCTA images from 235 eyes of 146 participants, divided into controls, diabetic patients without diabetic retinopathy (NoDR), and patients with nonproliferative diabetic retinopathy (NPDR). Skeletonized vascular maps were generated for the superficial (SCP) and deep capillary plexus (DCP). Vessel skeleton density (VSD), End Node (EN) density, Junction Node (JN) density, and Fractal Dimension (FD) were calculated in foveal, parafoveal, and total regions. Statistical comparisons were performed using linear mixed-effects models. EN density exhibited an inverted-U-shaped pattern across groups in both plexuses. VSD, JN density, and FD differed between groups, with lower values in NoDR and NPDR compared to controls. Discrimination analyses indicated that VSD performed better in differentiating NPDR from controls and NoDR, whereas JN density and FD were comparable for differentiating NoDR from controls. After adjusting for VSD across study groups, EN density in both layers and JN density in the SCP remained significant. Vascular metrics correlated with diabetes duration and glycemic level. EN and JN density emerge as promising biomarkers alongside VSD and FD for characterizing microvascular alterations in diabetic eyes, as topology-based parameters may provide information independent of vessel density.

**Supplementary Information:**

The online version contains supplementary material available at 10.1038/s41598-026-50339-y.

## Introduction

Fluorescein angiography, the gold standard for retinal imaging, is invasive and has limitations in demonstrating capillaries, the primary site of vascular damage in diabetes^1,2^. These shortcomings paved the way for the development of OCT angiography (OCTA). OCTA may be able to detect early and preclinical stages of diabetes due to its sufficient resolution in displaying capillaries^3^. Moreover, its non-invasive nature and potential for routine clinical use could facilitate the extraction of quantitative vascular features for future applications in automated classification, longitudinal follow-up, and telemedicine-based screening of patients^4,5^.

Following the commercialization of OCTA devices, numerous studies have sought to quantitatively analyze these images, particularly in diabetic patients. Several studies have attempted to determine in which retinal layer microvascular changes occur first and whether quantitative parameters can capture early changes at the preclinical stage. Studies have had mixed findings regarding the location of onset of early microvascular changes and perfusion trends in the NoDR stage^6–12^.

Quantitative OCTA parameters are generally divided into several categories: perfusion or density indices, FAZ-based measures, morphological descriptions, and choriocapillaris flow-deficit parameters. Evidence supporting significance FAZ differences between diabetic retinopathy (DR) stages and control groups appear more robust than that observed in NoDR comparisons^6,13^. Among perfusion-based indices, Geometric Perfusion Deficiency (GPD) appears promising in identifying preclinical DR, although its dependence on an arbitrary distance threshold may limit its reproducibility between different studies^8,14^.

Comparatively fewer studies have investigated retinal vascular morphology and analysis of its network topology. These studies have examined parameters such as tortuosity and fractal dimension (FD), the former increasing and the latter decreasing during the clinical stages of DR^15–17^.

This study analyzes the macular vascular network topology using End-Node (EN) and Junction-Node (JN) densities. We also evaluated the discrimination power of JN density and compared it against vessel skeleton density (VSD) and fractal dimension (FD).

## Methods

### Study design and participants

This is a cross-sectional observational study conducted from October 2024 to August 2025 in Torfeh Hospital, Tehran. Patients were divided into three groups: Control (*n* = 112 eyes), NoDR (*n* = 58 eyes), and NPDR (*n* = 65 eyes), with the latter comprising 20 mild, 29 moderate, and 17 severe cases of non-proliferative retinopathy. The primary demographic and clinical information for each group, including age, sex distribution, smoking status, BMI, diabetes duration, hypertension, HbA1c, and axial length is summarized in Table 1. Patients older than 18 years of age with type 2 diabetes were included in the study. Exclusion criteria were: diabetic macular edema, history of vitreoretinal surgery or macular photocoagulation, clinically significant epiretinal membrane (ERM) or vitreomacular traction (VMT), ocular comorbidities such as glaucoma, uveitis, optic neuropathy, concomitant retinal vascular diseases (e.g., retinal vein occlusion, hypertensive retinopathy), recent intraocular injection (within three months), visual acuity < 20/40, refractive error outside ± 4 diopters, pregnancy, significant media opacity, or poor-quality OCTA imaging. A total of 235 eyes from 146 patients were included. All patients provided written informed consent. This study adhered to the ethical principles of the Declaration of Helsinki and was approved by the Institutional Review Board of Shahid Beheshti University of Medical Sciences (Ethics ID: IR.SBMU.RETECH.REC.1403.333).

**Table 1:** Participant Demographics And Clinical Characteristics.

	Control	NoDR	NPDR	*P* Value
Eye, Subjects	112, 67	58, 34	65, 45	-
Age (Years)	65.4 ± 11	63.8 ± 11	63.7 ± 12.4	0.414
Gender (Male Percentage)	45%	48%	43%	0.866
Smoking Status	34%	33%	24%	0.348
BMI	25.08 ± 1.64	25.70 ± 2.34	25.70 ± 3.18	0.307
DM Duration (Years)	-	8.6 ± 4.9	14.7 ± 6.7	< 0.001
HTN	35%	30%	23%	0.213
HbA1C	-	7.7 ± 1.1	8.3 ± 0.9	< 0.001
Axial Length (mm)	22.97 ± 0.7	23.35 ± 0.85	23.10 ± 0.90	0.144

*DM* Diabetes Mellitus, *Hba1c* Hemoglobin A1c, *NoDR* Diabetes Without Retinopathy, *NPDR* Non-Proliferative Diabetic Retinopathy.

### OCTA imaging and segmentation protocol

OCTA images were acquired using a Topcon swept-source system (DRI OCT-1, Triton, Topcon, Tokyo, Japan) with 3 × 3 mm macular scans performed by an experienced operator. Segmentation of the superficial capillary plexus (SCP) and deep capillary plexus (DCP) was performed using IMAGEnet6 software (version 1.05E, Topcon Corporation, Tokyo, Japan; available at: topconhealthcare.eu/en_EU/products/imagenet-6)^17^. The SCP defined from the internal limiting membrane (ILM) to 15.6 μm below the IPL–INL junction, and the DCP between 15.6 and 70.2 μm below the IPL–INL junction. Images with motion, projection, or shadowing artifacts were excluded. Remaining images were exported at a resolution of 320 × 320 pixels.

### FAZ segmentation

To ensure reproducibility and address concerns regarding manual FAZ delineation, we implemented an automated approach based on edge detection, morphological processing, and connected component analysis in Python, as previously described by Díaz et al.^18^ The resulting FAZ masks were excluded from all subsequent vascular density and network analyses.

### Vascular segmentation and skeletonization

Vascular segmentation was performed using the Trainable Weka Segmentation plugin (Fiji, version 4.0). The classifier was trained by an experienced grader (S.K.) on ten representative images per group, classifying pixels into vascular and background categories. SCP and DCP images were subsequently processed in Fiji. Outliers and mis-segmented areas were corrected manually by MZ using brush tool. To ensure segmentation consistency, a subset of 15 images (five randomly selected from each study group, including both SCP and DCP layers) was used to quantitatively validate the segmentation using Dice similarity coefficient and Jaccard index (see Supplementary Table S1).

Probability maps from Weka were then denoised using the default Despeckle filter and converted to binary images using Otsu global thresholding in Fiji. Skeletonization was applied to the binary images to generate skeleton maps for topological analysis. The FAZ mask was then applied to exclude the foveal avascular zone.

### Node classification and quantitative analysis

Skeletonized images were analyzed for ENs, JNs, and branch segments. EN pixels were defined as those with only one adjacent pixel within a 3 × 3 neighborhood, branch segments with two adjacent pixels, and JN pixels with three or more adjacent pixels. Junction nodes with four adjacent pixels were excluded as potential crossover artifacts. Branches ≤ 3 pixels in length were removed only for visualization purposes and were retained in all quantitative analyses, as they likely represented true microvascular remnants affected by 2D segmentation rather than noise. This threshold was chosen empirically to retain true microvascular remnants while removing visualization noise.

Ocular magnification was corrected individually for each eye using the modified Bennett formula, based on axial length measurements obtained with the IOLMaster 700 (Carl Zeiss Meditec, Jena, Germany)^19^. Two concentric circles with nominal diameters of 1.5 mm and 2.5 mm were scaled accordingly and centered on the FAZ. The area between the FAZ and the corrected 1.5-mm circle was defined as the fovea, and the area between the two corrected circles as the parafovea.

### Fractal dimension (FD) calculation

FD was calculated using the conventional Box-Counting method. Skeletonized images were covered using hypothetical boxes of varying sizes (ε) and for each ε, the number of boxes containing at least one vascular pixel (N) was counted. Finally, FD was calculated as the slope of a logarithmic plot of the number of boxes N(ε) versus log(1/ε).

$$\:FD=li{m\frac{logN\left(\epsilon\:\right)}{\mathrm{l}\mathrm{o}\mathrm{g}\left(\frac{1}{\epsilon\:}\right)}}_{\epsilon\:\to\:0}$$

### Vessel skeleton density (VSD) calculation

VSD was defined as the percentage of vascular pixels in the skeletonized images relative to the total number of pixels within each region of interest. For each layer, VSD was calculated in the foveal, parafoveal, and total regions by dividing the number of skeletonized vascular pixels by the total area of the respective region.

### Statistical analysis

Statistical analyses were performed using SPSS v.26.0. Groups were compared using the Chi-square test for categorical data and One-way ANOVA for continuous variables (age, BMI, DM duration, HbA1c, and axial length). To account for intra-subject correlation between eyes, linear mixed-effects models with a random intercept for subject ID were used to analyze OCTA parameters (EN density, JN density, VSD, and FD). Post-hoc pairwise comparisons were conducted using estimated marginal means (EMMs) with Bonferroni correction. Subgroup analyses within the NPDR group were planned; however, comparisons between mild and moderate NPDR were not performed, as all p-values were 1.0, reflecting minimal variability in this limited sample size. Interpretations of subgroup results should therefore be made with caution. Detailed statistical performance of VSD, JN density, and FD in differentiating NPDR severity levels, along with the results of subgroup pairwise comparisons within the NPDR group, are provided in Supplementary Tables S4 and S5, respectively.

Area Under the Curve (AUC) with 95% CIs were used to evaluate discriminatory performance. AUC values were interpreted as follows: 0.50–0.60 = failure, 0.61–0.70 = poor, 0.71–0.80 = fair, 0.81–0.90 = considerable, and 0.91–1.00 = excellent^20^. A generalized linear model was used to derive adjusted topological metrics, accounting for variations in VSD. Also Spearman correlation was applied to assess the relationship between OCTA metrics and systemic variables (DM duration, HbA1c). A p-value < 0.05 was considered statistically significant.

## Results

### Demographic and clinical characteristics

A total of 235 eyes from 146 individuals were included in the study and were divided into three groups: control, NoDR, and NPDR (Table 1). The distribution of qualitative variables including gender, smoking status, and frequency of hypertension did not differ significantly between the three groups. (all *p* > 0.05) Among quantitative variables, there were no differences in age, BMI, or axial length among the groups. As expected, diabetes duration and HbA1C were higher in the NPDR group than in the NoDR group. (*p* < 0.001) Therefore, the study groups were considered comparable in terms of demographic and ocular characteristics.

### Vascular network EN and JN densities

In the SCP and DCP, both EN and JN densities differed significantly across all regions. (with the exception of EN density in the DCP fovea) (all *p* < 0.05; Table 2). Post-hoc analyses revealed a consistent pattern of EN density alteration across regions. In the NoDR group, EN density was significantly higher in the SCP fovea and DCP parafovea and total regions compared to controls (*p* < 0.05). Conversely, the NPDR group demonstrated lower EN density than the NoDR group. Notably, similar trends towards significance were observed in the DCP fovea (*p* = 0.118) and SCP parafovea and total regions (*p* = 0.067–0.075), suggesting a widespread remodeling effect (Supplementary Table S2).

**Table 2:** Quantitative Measurements In Each Group.

		VSD (%)		EN Density (mm⁻²)		JN Density (mm⁻²)		FD	
		SCP	DCP	SCP	DCP	SCP	DCP	SCP	DCP
Fovea	Control	13.5 ± 1.1	12.0 ± 1.4	110 ± 14	108 ± 12	92 ± 21	90 ± 22	1.38 ± 0.06	1.35 ± 0.07
	NoDR	13.1 ± 1.4	11.5 ± 1.8	118 ± 16	114 ± 16	85 ± 28	78 ± 25	1.32 ± 0.07	1.35 ± 0.07
	NPDR	11.8 ± 1.5	10.7 ± 1.8	98 ± 19	110 ± 20	74 ± 26	69 ± 25	1.30 ± 0.08	1.27 ± 0.10
	P value	< 0.001***	< 0.001***	< 0.001***	0.113	< 0.001***	< 0.001***	< 0.001***	< 0.001***
Parafovea	Control	15.5 ± 1.1	15.8 ± 0.9	102 ± 11	101 ± 11	169 ± 21	169 ± 21	1.42 ± 0.03	1.42 ± 0.03
	NoDR	15.3 ± 1.4	15.2 ± 1.2	107 ± 15	108 ± 14	157 ± 29	157 ± 29	1.42 ± 0.04	1.42 ± 0.05
	NPDR	14.1 ± 1.5	13.9 ± 1.4	100 ± 12	107 ± 14	134 ± 30	134 ± 30	1.40 ± 0.04	1.39 ± 0.03
	P value	< 0.001***	< 0.001***	0.011*	0.005**	< 0.001***	< 0.001***	< 0.001***	< 0.001***
Total	Control	15.0 ± 1.1	15.4 ± 1.0	106 ± 10	109 ± 10	149 ± 24	160 ± 21	1.61 ± 0.01	1.61 ± 0.01
	NoDR	14.7 ± 1.4	14.9 ± 1.2	111 ± 13	114 ± 12	145 ± 31	151 ± 27	1.60 ± 0.02	1.61 ± 0.04
	NPDR	13.7 ± 1.3	13.8 ± 1.3	103 ± 11	112 ± 12	128 ± 28	132 ± 26	1.58 ± 0.02	1.59 ± 0.02
	P value	< 0.001***	< 0.001***	0.001**	0.042*	< 0.001***	< 0.001***	< 0.001***	< 0.001***

* < 0.05, ** < 0.01, *** < 0.001, *EN: VSD* Vessel Skelton Density, End Node; *JN* Junction Node; *SCP* Superficial Capillary Plexus, *DCP* Deep Capillary Plexus, *FD* Fractal Dimension.

JN density exhibited a constant decreasing trend across the three groups in both SCP and DCP layers (all *p* < 0.001). Significant lower JN densities were evident in the NoDR group compared with controls in the SCP foveal region (85 ± 28 vs. 92 ± 21 mm⁻², *p* = 0.004), the DCP parafoveal region (157 ± 29 vs. 169 ± 21 mm⁻², *p* = 0.018), and the DCP total region (151 ± 27 vs. 160 ± 21 mm⁻², *p* = 0.039). JN density discrimination between NoDR and Control was failure to poor (AUC = 0.53–0.63) and between NPDR and NoDR was poor to fair (AUC = 0.60–0.72). The discrimination between NPDR and control subjects was fair to considerable (AUC = 0.71–0.84, Table 3).

**Table 3:** Pairwise comparisons between study groups.

			VSD		JN Density		FD	
			SCP	DCP	SCP	DCP	SCP	DCP
NoDR vs Control	Fovea	P Value	0.103	0.142	0.004**	0.077*	<0.001***	1.000
		AUC (CL 95%)	0.59 (0.50–0.68)	0.58 (0.48–0.67)	0.63 (0.54–0.72)	0.60 (0.51–0.70)	0.77 (0.69–0.85)	0.66 (0.43–0.61)
	Parafovea	P Value	0.798	0.009**	1.000	0.018*	1.000	1.000
		AUC (CL 95%)	0.52 (0.43–0.62)	0.64 (0.56–0.73)	0.53 (0.42–0.62)	0.63 (0.54–0.73)	0.55 (0.46–0.65)	0.56 (0.47–0.66)
	Total	P Value	0.335	0.023*	0.833	0.039*	0.757	0.644
		AUC (CL 95%)	0.55 (0.45–0.64)	0.63 (0.53–0.72)	0.54 (0.44–0.63)	0.62 (0.53–0.71)	0.57 (0.48–0.67)	0.62 (0.53–0.72)
NPDR vs Control	Fovea	P Value	<0.001***	<0.001***	<0.001***	<0.001***	<0.001***	<0.001***
		AUC (CL 95%)	0.81 (0.75–0.88)	0.71 (0.63–0.79)	0.80 (0.73–0.87)	0.71 (0.63–0.79)	0.79 (0.72–0.86)	0.75 (0.68–0.83)
	Parafovea	P Value	<0.001***	<0.001***	<0.001***	<0.001***	0.002**	<0.001***
		AUC (CL 95%)	0.79 (0.72–0.86)	0.87 (0.81–0.93)	0.74 (0.66–0.81)	0.84 (0.78–0.90)	0.69 (0.61–0.77)	0.73 (0.66–0.81)
	Total	P Value	<0.001***	<0.001***	<0.001***	<0.001***	<0.001***	<0.001***
		AUC (CL 95%)	0.79 (0.72–0.86)	0.84 (0.78–0.91)	0.73 (0.66–0.81)	0.82 (0.75–0.88)	0.82 (0.75–0.88)	0.85 (0.79–0.91)
NPDR vs NoDR	Fovea	P Value	<0.001***	0.054	0.002**	0.202	<0.001***	<0.001***
		AUC (CL 95%)	0.74 (0.65–0.82)	0.63 (0.54–0.73)	0.66 (0.57–0.76)	0.60 (0.51–0.70)	0.56 (0.46–0.66)	0.73 (0.65–0.82)
	Parafovea	P Value	<0.001***	<0.001***	0.007**	<0.001***	0.010*	0.001**
		AUC (CL 95%)	0.72 (0.63–0.81)	0.76 (0.68–0.84)	0.68 (0.59–0.77)	0.72 (0.64–0.81)	0.64 (0.55–0.74)	0.66 (0.56–0.76)
	Total	P Value	<0.001***	<0.001***	0.011*	0.004**	<0.001***	<0.001***
		AUC (CL 95%)	0.71 (0.62–0.80)	0.74 (0.66–0.83)	0.66 (0.56–0.76)	0.70 (0.61–0.79)	0.75 (0.67–0.83)	0.69 (0.60–0.78)

* < 0.05, ** < 0.01, *** < 0.001, *VSD* Vessel Skeleton Density, *JN* Junction Node, *FD* Fractal Dimension, *SCP* Superficial Capillary Plexus, *DCP* Deep Capillary Plexus.

### Fractal dimension

FD in both SCP and DCP revealed a significant decreasing trend among the three groups. (SCP, DCP: *p* < 0.001, Table 2). Post-hoc pairwise comparisons indicated that FD reductions in NoDR vs. control was only significant in SCP fovea regions (1.32 ± 0.07 vs. 1.38 ± 0.06, *p* < 0.001). NPDR group had a significantly lower FD compared to NoDR in all regions (*p* ≤ 0.01, Table 3). Discrimination between the two consecutive groups ranged from failure to fair (0.55–077.55). FD had fair to considerable discrimination between NPDR and control (0.69–0.85, Table 3).

### Vascular skeleton density

In both layers, VSD showed a significant decreasing trend across the three study groups (all *p* < 0.001). In pairwise comparison between the NoDR and Control groups, the NoDR group had significantly lower values in the parafovea and total regions of the DCP layer (parafovea: 15.2 ± 1.2% vs. 15.8 ± 0.9%, *p* = 0.009; total: 14.9 ± 1.2% vs. 15.4 ± 1.0%, *p* = 0.023). Almost all other pairwise comparisons were also significant (*p* < 0.001; Table 3).

### Comparison between JN density, FD and VSD

JN density, FD and VSD demonstrated fair to considerable discriminatory ability between NPDR and control groups in both layers. However, VSD significantly outperformed both JN and FD. All three parameters showed comparable performance in differentiating control from NoDR. In the NPDR vs. NoDR comparison, VSD again yielded significantly better discrimination than JN density in SCP and parafoveal region of DCP, whereas FD and JN densities exhibited similar performance. A detailed quantitative comparison of their discriminatory performance is provided in Supplementary Table S3.

### VSD-adjusted analysis of topological metrics

After adjustment for VSD using a univariate general linear model, EN density remained significantly different across study groups in all regions and layers (data not shown, *p* < 0.01 in SCP and *p* < 0.05 in DCP). JN density also showed significant group differences in SCP; however, this effect was not observed in the DCP.

### Interrelationship between topological metrics and VSD

Interestingly, a significant inverse correlation between JN and EN densities was observed within the NoDR group, particularly in the parafoveal and total regions of both plexuses (SCP: *r* = −0.342 and − 0.298, *p* = 0.007 and 0.020, respectively; DCP: *r* = −0.347 and − 0.305, *p* = 0.006 and 0.017, respectively). A similar inverse trend was noted in the control group. In contrast, the NPDR group exhibited a shift towards a positive correlation between these parameters, reaching significance in the SCP total region (*r* = 0.245, *p* = 0.039) and the DCP fovea (*r* = 0.355, *p* = 0.002).

Spearman correlation analysis revealed a strong positive association between JN and VSD across all regions and both plexuses (r ranging from 0.91 to 0.94, *p* < 0.001). Conversely, EN density showed a mild to moderate negative correlation with VSD, which was significant in the parafoveal and total areas of the DCP (r ranging from − 0.32 to − 0.37, *p* < 0.001) and weaker in the corresponding regions of the SCP (*r* = − 0.193, *p* = 0.003 for parafovea; *r* = − 0.114, *p* = 0.079 for total).

### Correlation with clinical parameters

Spearman correlation analysis revealed significant negative associations between OCTA parameters and both diabetes duration and HbA1c. In the SCP, VSD, JN density, and FD all showed significant negative correlations with diabetes duration (r ranging from − 0.244 to −0.429, *p* = 0.01). Similarly, in the DCP total region, all three parameters demonstrated significant negative correlations with duration (r ranging from − 0.382 to −0.414, *p* < 0.001). Regarding HbA1c, significant negative correlations were observed in the SCP (r ranging from − 0.194 to −0.279, *p* = 0.05) and the DCP total region (r ranging from − 0.240 to −0.259, *p* = 0.01).

## Discussion

In this study, we examined four parameters related to the retinal vascular network: EN density, JN density, and FD along with VSD. First, the analysis was performed between the three groups: control, NoDR, and NPDR. Then, in a separate analysis, we compared the aforementioned parameters with each other to determine their ability to discriminate between the groups. We attempted to determine whether the retinal vascular network topology undergoes changes before the onset of the clinical stage. We further assessed whether the topological parameters of the retinal vascular network merely reflect vessel density changes or provide independent information.

An inverted-U pattern was observed in EN density changes across the three different groups, in each of the SCP and DCP layers (Fig. 2). In contrast, JN density, FD, and VSD showed consistently lower values across these groups. Several factors could potentially explain the opposite pattern observed between EN density and JN density in our OCTA scans. We hypothesize that early vascular remodeling may occur secondary to obstruction in the capillary network, leading the connections in the retinal vascular network reduced and converting connective branch and JNs into terminal branches and ENs. Consequently, a decrease in the JN density is accompanied by a reciprocal increase in EN density. In contrast, during the clinical stage of DR, obstruction occurs in the more proximal branches and at the level of the arterioles or venules, resulting in the complete loss of the distal branches and a concurrent reduction in both EN and JN densities. This explanation aligns with the report by Rosen et al., who stated that early changes in NoDR can be detected through an analysis of parafoveal capillary density^11^. The proposed mechanism is illustrated in Fig. 3.

It is critical to recognize that the retinal vascular network is inherently three-dimensional. Unlike renal nephrons (where terminal loops terminate in glomeruli), no analogous structure exists in the retina. ENs derived from two-dimensional OCTA images cannot be interpreted as true alterations in the three-dimensional vascular architecture. Therefore, EN density interpretation must account for characteristics of OCTA acquisition and image processing. In our images, weak signals from narrow connecting branches or vessel orientation may cause portions of the network to appear as dead ends (“End Nodes”). This may explain EN prevalence: reduced perfusion in weaker terminal branches could lead to their segmentation during imaging/image processing, manifesting as elevated EN density. Additionally, vertical anastomoses from the SCP to the intermediate capillary plexus and DCP may contribute to ENs in our images^21^. We propose that these factors collectively influence EN formation in OCTA images. However, determining their relative contributions requires matching 2D and 3D imaging data.

Yu et al. did not observe a significant difference in EN density between control and NoDR groups in their study^22^. However, they also reported an increasing trend. Also, they noted significantly higher EN densities in mild NPDR in the DCP layer compared to control group. A significant distinction exists between the EN and JN density calculations in our study and that of Yu et al., despite the similar nomenclature used. Yu et al. reported EN values in two forms: “EN number” and “EN density.” The “EN number” is an unnormalized parameter, which inherently limits its comparability between different eyes and across studies. The “EN density,” defined as EN number divided by vascular length, is a structurally normalized parameter that we believe may be susceptible to ratio-driven bias. As the researchers themselves have noted, observed changes in EN density in their study did not necessarily reflect changes in EN number, but demonstrated variations in vascular length. In other words, the basis for this normalization (vascular length) is itself a disease-dependent and potentially unstable variable. In contrast, our study calculated EN density based on a normalized area of mm², which was further adjusted for axial length (AL). We contend that this method of calculation provides a more robust basis for statistical comparison, allowing for clearer insights into actual changes in ENs independent of vascular length fluctuations. These calculations suggest that, regardless of possible changes in vascular length, EN density increases as a separate variable, reflecting vascular network topological alterations in the early stages of diabetes.

We found several significant differences between the NoDR and Control groups. Specifically, both JN density and VSD were significantly lower in the NoDR group compared to the Control group within the DCP layer, particularly in the parafoveal and total regions (Table 3). This finding indicates preclinical alterations that precede the clinical stage. Furthermore, JN density, FD, and VSD were significantly lower in the NPDR group compared to the Control group across all comparisons.

FD was significantly lower in the foveal region of the SCP in the NoDR group compared to controls, while no significant differences were observed in other regions. Similarly, Hogg et al. reported a significant FD reduction in patients with overt DR compared to healthy controls, but found no difference between NoDR and control eyes, which aligns with our findings^23^. These results are consistent with studies by Zahid et al. and Hashmi et al., who reported significantly lower FD in clinical stages of DR compared to healthy individuals^24,25^. In our study, FD demonstrated higher diagnostic performance (AUC up to 0.85 for NPDR vs. healthy controls) compared with Hogg et al., who reported a maximum AUC of 0.61. Unlike our approach, which applied box-counting on fully skeletonized 3 × 3-mm images, highly sensitive to early microvascular branching loss, their analyses employed Fourier and multifractal methods on non-skeletonized wider-field (10°×10°) scans, which are more influenced by vessel caliber and overall density.

Our analysis indicated that VSD demonstrates a superior discriminatory capability compared to both JN density and FD. Although all parameters showed comparable effectiveness in distinguishing between the NoDR and Control groups (Supplementary Table S3), VSD significantly outperformed JN density and FD in differentiating NPDR from NoDR, and NPDR from Control. JN density’s performance was consistently comparable to that of FD across the majority of evaluations. This is consistent with the findings of Mendes et al., who likewise reported a higher AUC for VSD than for FD^26^. The AUC ranges observed in our study were comparable to their reported values, although Mendes et al. showed relatively lower DCP sensitivity, which may again reflect differences in OCTA acquisition protocols or image-processing methods. (SCP: 0.79 vs. 0.85; DCP: 0.84 vs. 0.65, in our study and Mendes’s data, respectively.)

In our study, the DCP layer showed greater sensitivity to vessel density (VSD) variations than the SCP layer, except in the foveal region, where the opposite pattern likely results from the less well-defined FAZ contour in DCP affecting the measured foveal area. The NoDR group demonstrated significantly lower VSD than controls within both parafoveal and total DCP regions, suggesting early microvascular compromise in DCP even before clinically detectable DR. These findings differ from Ong et al., who reported significant VSD changes only at the clinical stage of DR and found SCP to be more sensitive than DCP^27^. As they noted, noise and projection artifacts in their DCP images may have limited sensitivity. In contrast, we excluded all such affected images and used different OCTA acquisition protocols, which likely enhanced DCP detection capability and enabled identification of subclinical changes. This is consistent with the observations of Meshi et al.^6^ and Simonett et al.^7^ The DCP’s unique architecture and hemodynamic environment may render it more vulnerable to hyperglycemic damage.

The JN density exhibited a strong and significant positive correlation with VSD across all of our analyses. In contrast, EN density showed an inverse relationship with VSD—as theoretically expected—but this association was either weak or statistically insignificant. Following adjustment for VSC, both EN and JN densities remained significantly different across the groups. Accordingly, we propose that EN and JN densities may not directly reflect retinal perfusion, but rather represents the topological characteristics of the vascular network, thereby providing complementary structural information about the retinal microvasculature.

Conversely, Spearman correlation revealed an inverse relationship between EN density and JN density in the Control and NoDR groups, whereas a positive correlation was observed in the NPDR group. This finding could imply an early compensatory mechanism operating in the preclinical phase, which diminishes upon the onset of clinical manifestation. However, given the cross-sectional nature of this study, any explanation remains theoretical.

JN density, FD, and VSD had a significant negative correlation with HbA1C and diabetes duration. Longitudinal studies are needed to determine how these parameters are associated with DR-related complications.

In our analysis, we excluded grade 4 JNs as vascular crossover points and examined their density separately. Also because EN exhibits a non-monotonic trajectory, its interpretation requires caution in early stages and should ideally be considered alongside JN density, FD and VSD.

### Limitations and future directions

This study has several limitations. Its cross-sectional design restricts definitive conclusions about the temporal sequence of vascular changes. Future longitudinal studies are essential to validate the observed inverted U-shaped pattern and the predictive value of these parameters for DR progression. Our sample size was modest and from a single center, necessitating external validation in larger cohorts to confirm generalizability. The image processing pipeline, while standardized, involves manual components; fully automated segmentation would enhance reproducibility. Furthermore, the interpretation of findings is limited by 2D imaging; validation with 3D OCTA or projection-resolved imaging is required to clarify the anatomical meaning of ENs, which may represent vertical anastomoses. The lack of evaluation of peripheral retinal blood flow is another limitation. Also, while demographic and lab data were available, diabetes duration was self-reported by patients without a specific dataset, potentially introducing bias into correlations with our parameters. This aspect of our findings requires cautious interpretation. Finally, the pathophysiological mechanisms underlying the observed topological changes, particularly the reversed correlation between JN and EN densities, warrant exploration through parallel histological or experimental models to understand this potential compensatory mechanism.

**Fig. 1:**
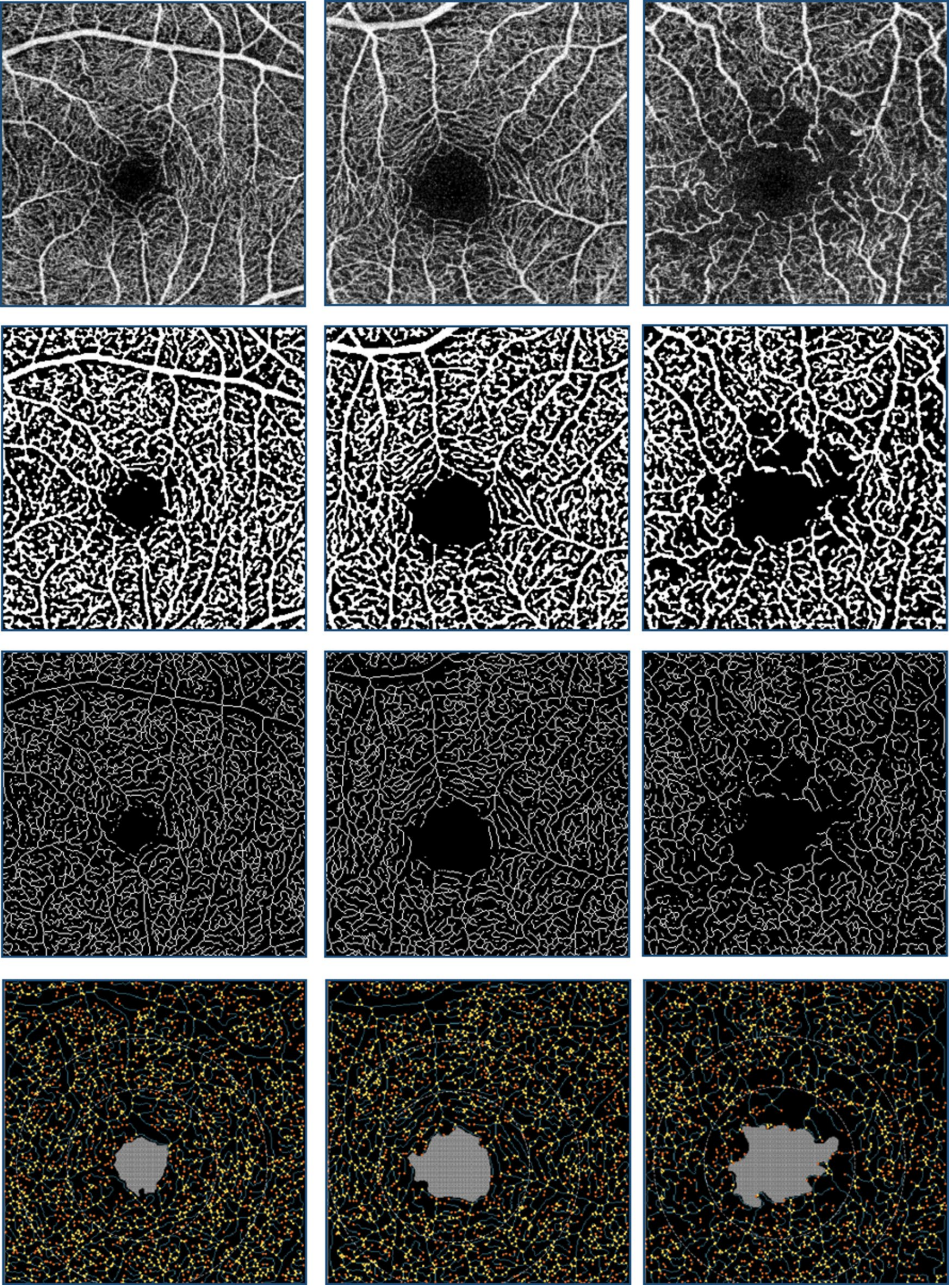
Sample processed images. The first row is the raw images of SCP, which from left are the control, NoDR, and NPDR groups. The second row is the binarized images. The third row is the skeletonized images, and the fourth row is a visualization of the analyzed vascular network map. Yellow dots correspond to junction nodes and brick-colored dots correspond to end nodes. The FAZ area is highlighted in gray, and the vessels are delineated with blue lines.

**Fig. 2:**
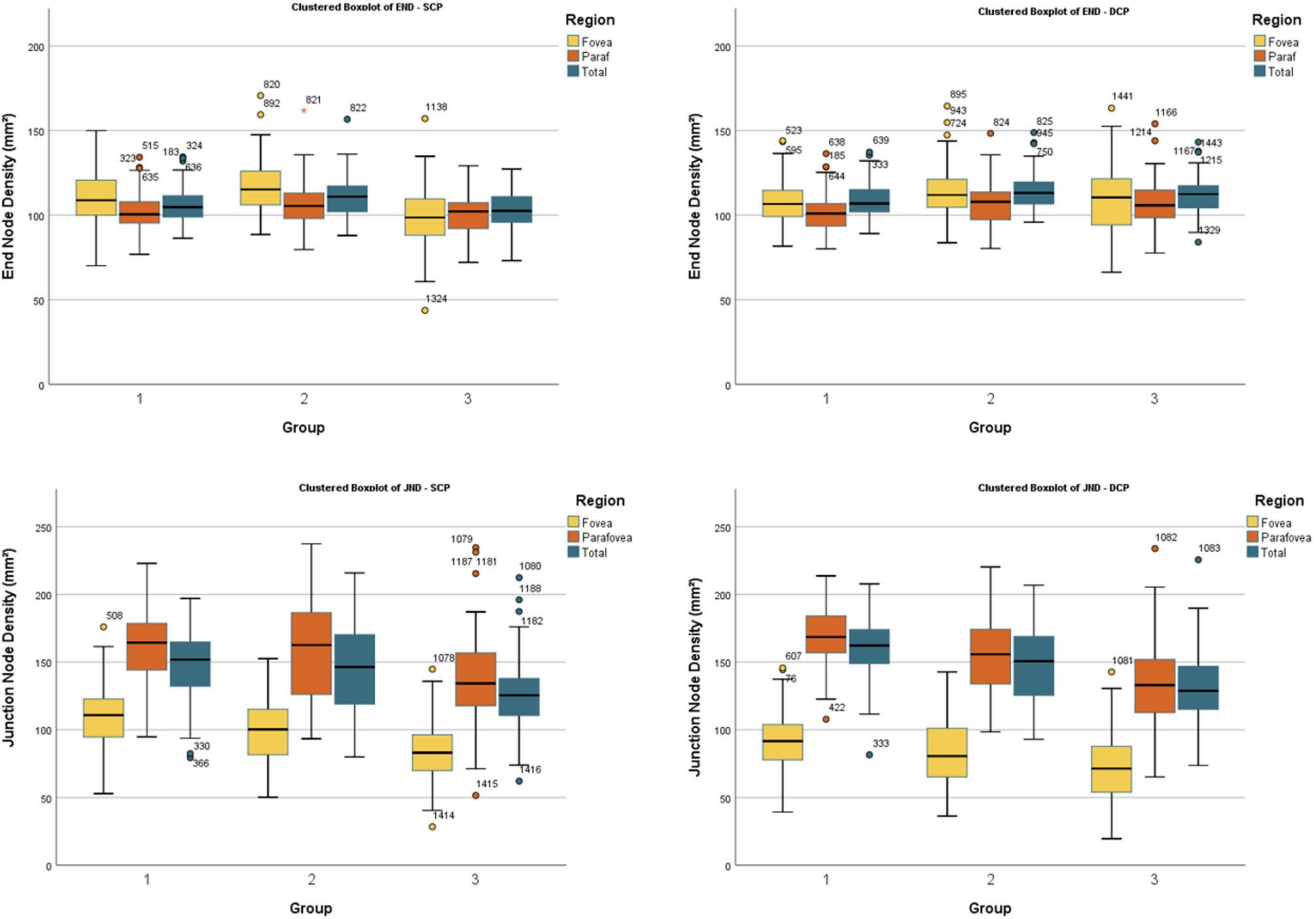
Boxplots illustrating End Node density, Junction Node Density, and Fractal Dimension distributions across study groups. The first row shows an inverted-U pattern in the EN Density changes in SCP and DCP layers. Group 1 = control group, Group 2 = diabetic patients without diabetic retinopathy (NoDR), and Group 3 corresponds = non-proliferative diabetic retinopathy (NPDR).

**Fig. 3:**
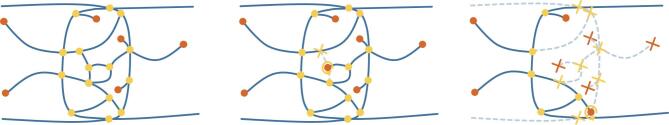
Schematic illustration of a possible explanation for JN and EN changes in OCTA 2D images. Left: A normal vascular network. Middle row: Following capillary occlusion, a connecting branch is lost. Subsequently one JN disappears completely, and an EN replaces a former JN in the new image (leading to increased EN Density and decreased JN Density). In the image on the right, following occlusion of a root branch, most of its distal branches are also occluded resulting in an overall decrease in both JN and EN density. Brick-colored Dots: EN, Yellow Dots: JN.

## Supplementary Information

Below is the link to the electronic supplementary material.

Supplementary material 1.

## Data Availability

The datasets generated during and/or analyzed during the current study are available from the corresponding author on reasonable request.

## Abbreviations

AUC: Area Under the Curve
BMI: Body Mass Index
DCP: Deep Capillary Plexus
DR: Diabetic Retinopathy
EN: End Node
ERM: Epiretinal Membrane
FAZ: Foveal Avascular Zone
FD: Fractal Dimension
GPD: Geometric Perfusion Deficit
HbA1c: Glycated Hemoglobin
IOP: Intraocular Pressure
JN: Junction Node
NoDR: Diabetes without Diabetic Retinopathy
NPDR: Nonproliferative Diabetic Retinopathy
OCTA: Optical Coherence Tomography Angiography
SCP: Superficial Capillary Plexus
VSD: Vessel Skeleton Density
VMT: Vitreomacular Traction
