# Supplementary material for: Opposing end-node and junction-node remodeling patterns in diabetic patients on optical coherence tomography angiography
Source: Sci Rep. 2026 Apr 25;16:19146. doi: 10.1038/s41598-026-50339-y (PMC13279989; doi:10.1038/s41598-026-50339-y)
Supplement: Supplementary file 1 — Supplementary material 1. [file 41598_2026_50339_MOESM1_ESM.docx]

| **Supplementary Table S1. Quantitative Validation Metrics for Weka Segmentation** | | |
| --- | --- | --- |
|  | Dice coefficient (Mean ± SD) | Jaccard index (Mean ± SD) |
| SCP | 0.999 ± 0.001 | 0.997 ± 0.002 |
| DCP | 0.998 ± 0.002 | 0.995 ± 0.004 |
| The high agreement values should be interpreted in the context of manual refinement of the automated segmentation. These metrics were used to confirm consistency rather than to establish independent ground truth validation. | | |

| Supplementary Table S2 – EN Density Pairwise Comparisons Between Study Groups | | | | | | | |
| --- | --- | --- | --- | --- | --- | --- | --- |
|  |  | NoDR vs Control | | NPDR vs Control | | NPDR vs NoDR | |
|  |  | SCP | DCP | SCP | DCP | SCP | DCP |
| Fovea | Mean + SD | 118±16 vs 110±14 | 114±16 vs 108±12 | 98±19 vs 110±14 | 110±20 vs 108±12 | 98±19 vs 118±16 | 110±20 vs 114±16 |
|  | P Value | 0.007** | 0.118 | <0.001*** | 0.801 | <0.001*** | 1.000* |
| Parafovea | Mean + SD | 107±15 vs 102±11 | 108±14 vs 101±11 | 100±12 vs 102±11 | 107±14 vs 101±11 | 100±12 vs 107±15 | 107±14 vs 108±14 |
|  | P Value | 0.067 | 0.020* | 1.000 | 0.018* | 0.010* | 1.000 |
| Total | Mean + SD | 111±13 vs 106±10 | 114±12 vs 109±10 | 103±11 vs 106±10 | 112±12 vs 109±10 | 103±11 vs 111±13 | 112±12 vs 114±12 |
|  | P Value | 0.075 | 0.044* | 0.121 | 0.374 | <0.001*** | 1.000 |
| * < 0.05, ** < 0.01, *** < 0.001, SCP = Superficial Capillary Plexus; DCP = Deep Capillary Plexus; | | | | | | | |

| Supplementary Table S3. Statistical Performance Of VSD, JN Density and FD In Differentiating Study Group | | | | | | | | |
| --- | --- | --- | --- | --- | --- | --- | --- | --- |
| NoDR vs Control |  |  | VSD vs JN Density | | VSD vs FD | | JN vs FD | |
|  |  |  | SCP | DCP | SCP | DCP | SCP | DCP |
|  | Fovea | AUC difference | -0.04 | -0.03 | 0.019 | 0.06 | -0.14 | 0.08 |
|  |  | P value | 0.140 | 0.211 | 0.001** | 0.364 | 0.014* | 0.176 |
|  | Parafovea | AUC difference | <0.01 | 0.01 | -0.028 | 0.08 | -0.03 | 0.07 |
|  |  | P value | 0.828 | 0.683 | 0.667 | 0.114 | 0.621 | 0.160 |
|  | Total | AUC difference | 0.01 | 0.01 | -0.03 | 0.001 | -0.04 | -0.005 |
|  |  | P value | 0.505 | 0.779 | 0.620 | 0.974 | 0.453 | 0.910 |
| NPDR vs Control | Fovea | AUC difference | 0.02 | -0.01 | 0.03 | -0.05 | 0.006 | -0.04 |
|  |  | P value | 0.209 | 0.780 | 0.545 | 0.364 | 0.896 | 0.411 |
|  | Parafovea | AUC difference | 0.06 | 0.03 | 0.10 | 0.4 | 0.04 | 0.11 |
|  |  | P value | <0.001*** | 0.064 | 0.032* | 0.002** | 0.370 | 0.02* |
|  | Total | AUC difference | 0.06 | 0.02 | -0.03 | -0.003 | -0.09 | -0.03 |
|  |  | P value | <0.001*** | 0.121 | 0.485 | 0.917 | 0.055 | 0.480 |
| NPDR vs NoDR | Fovea | AUC difference | 0.08 | 0.03 | 0.17 | -0.10 | 0.09 | -0.13 |
|  |  | P value | <0.001*** | 0.149 | 0.005** | 0.070 | 0.147 | 0.016* |
|  | Parafovea | AUC difference | 0.04 | 0.039 | 0.08 | 0.10 | 0.03 | 0.05 |
|  |  | P value | 0.05 | 0.55 | 0.156 | 0.052 | 0.576 | 0.281 |
|  | Total | AUC difference | 0.010 | 0.149 | -0.04 | 0.05 | -0.01 | -0.01 |
|  |  | P value | 0.002** | 0.043* | 0.460 | 0.103 | 0.114 | 0.951 |
| * < 0.05, ** < 0.01, *** < 0.001, VSD: Vessel Skeleton Density; JN = Junction Node; FD = Fractal Dimension, SCP = Superficial Capillary Plexus; DCP = Deep Capillary Plexus | | | | | | | | |

| Supplementary Table S4. Statistical Performance of VSD, JN Density and FD in Differentiating NPDR Severity | | | | | | | | | |
| --- | --- | --- | --- | --- | --- | --- | --- | --- | --- |
|  |  | VSD (%) | | EN Density (mm⁻²) | | JN Density (mm⁻²) | | FD | |
|  |  | SCP | DCP | SCP | DCP | SCP | DCP | SCP | DCP |
| Fovea | Mild | 12.4 ± 1.4 | 11.1 ± 2.1 | 108±18 | 107±20 | 88±25 | 80±31 | 1.31 ± 0.07 | 1.28 ± 0.11 |
|  | Moderate | 12.0 ± 1.4 | 10.8 ± 1.6 | 96±19 | 113±21 | 90±21 | 64±20 | 1.32 ± 0.07 | 1.28 ± 0.09 |
|  | Severe | 10.7 ± 1.4 | 10.2 ± 1.5 | 89±16 | 110±20 | 67±17 | 64±20 | 1.25 ± 0.08 | 1.24 ± 0.09 |
|  | P value | 0.001** | 0.324 | 0.708 | 0.500 | 0.005** | 0.139 | 0.005** | 0.228 |
| Parafovea | Mild | 14.6 ± 1.2 | 14.4 ± 1.5 | 105±9 | 106±13 | 141±33 | 140±34 | 1.40 ± 0.03 | 1.40 ± 0.04 |
|  | Moderate | 14.41 ± 1.5 | 14.1 ± 1.3 | 101±12 | 109±16 | 146±34 | 140±27 | 1.40 ± 0.04 | 1.39 ± 0.03 |
|  | Severe | 13.0 ± 1.4 | 13.1 ±1.1 | 92±11 | 106±13 | 117±26 | 117±24 | 1.37 ± 0.04 | 1.38 ± 0.04 |
|  | P value | 0.004** | 0.031* | 0.009** | 0.708 | 0.006** | 0.056 | 0.034* | 0.109 |
| Total | Mild | 14.2 ± 1.1 | 14.2 ± 1.4 | 107±10 | 111±10 | 132±28 | 139±31 | 1.59 ± 0.02 | 1.59 ± 0.02 |
|  | Moderate | 13.9 ± 1.3 | 13.9 ± 1.3 | 104±11 | 113±14 | 133±29 | 135±24 | 1.59 ± 0.02 | 1.59 ± 0.02 |
|  | Severe | 12.8 ± 1.2 | 12.9 ± 0.9 | 96±8 | 111±11 | 111±20 | 117±17 | 1.57 ± 0.02 | 1.57 ± 0.02 |
|  | P value | 0.003** | 0.069 | 0.019* | 0.831 | 0.031* | 0.035 | <0.001*** | 0.001** |
| * < 0.05, ** < 0.01, *** < 0.001, VSD: Vessel Skeleton Density; EN: End Node; JN = Junction Node; FD = Fractal Dimension, SCP = Superficial Capillary Plexus; DCP = Deep Capillary Plexus | | | | | | | | | |

| Supplementary Table S5. P values for Subgroup Pairwise Comparison Analysis in NPDR Group | | | | | | | | |
| --- | --- | --- | --- | --- | --- | --- | --- | --- |
|  |  | VSD | | JN Density | | FD | |  |
|  |  | SCP | DCP | SCP | DCP | SCP | DCP |  |
| Severe vs Mild | Fovea | 0.011* | 0.405 | 0.014 | 0.145 | 0.011* | 0.580 |  |
|  | Parafovea | 0.004** | 0.032* | 0.022 | 0.092 | 0.062 | 0.115 |  |
|  | Total | 0.002** | 0.069 | 0.048* | 0.037* | 0.001** | 0.001 |  |
| Severe vs Moderate | Fovea | 0.008** | 1.000 | 1.000 | 0.526 | 0.008** | 0.227 |  |
|  | Parafovea | 0.014* | 0.090 | 1.000 | 0.085 | 0.053 | 0.373 |  |
|  | Total | 0.002** | 0.219 | 1.000 | 0.106 | 0.001** | 0.005** |  |
| * < 0.05, ** < 0.01, *** < 0.001, VSD: Vessel Skeleton Density; JN = Junction Node; FD = Fractal Dimension, SCP = Superficial Capillary Plexus; DCP = Deep Capillary Plexus | | | | | | | | |
